# The Matrix Metalloproteases and Endothelin-1 in Infection-Associated Preterm Birth

**DOI:** 10.1155/2010/657039

**Published:** 2010-07-26

**Authors:** Nicole S. Olgun, Sandra E. Reznik

**Affiliations:** Department of Pharmaceutical Sciences, College of Pharmacy and Allied Health Professions, St. John's University, St. Albert Hall G018-B, 8000 Utopia Parkway, Jamaica, NY 11439, USA

## Abstract

Preterm birth (PTB) is clinically defined as any delivery which occurs before the completion of 37 weeks of gestation, and is currently the most important problem in obstetrics. In the United States, PTB accounts for 12-13% of all live births, and, with the exception of fetuses suffering from anomalies, is the primary cause of perinatal mortality. While the risk factors for PTB are numerous, the single most common cause is intrauterine infection. As there is currently no FDA-approved therapy for infection-associated PTB, understanding the pathogenesis of preterm labor (PTL) and delivery should be given high priority. The matrix metalloproteinases (MMPs) are a family of enzymes that have been implicated in normal parturition as well as infection-triggered rupture of membranes and preterm birth. Several lines of evidence also suggest a role for endothelin-1 (ET-1) in infection-associated preterm delivery. This paper focuses on the evidence that the MMPs and ET-1 act in the same molecular pathway in preterm birth.

## 1. Introduction

Preterm birth (PTB) is defined as any delivery occurring before the completion of 37 weeks of gestation, and currently accounts for 12-13% of all births in the United States. Despite advances that have been made in the field of neonatology and in our understanding of the mechanisms involved in parturition, the occurrence of PTB has not declined, and accounts for approximately 5,000 infant deaths in the United States per year [1]. In 2005, the National Center for Health Statistics (Centers for Disease Control and Prevention) reported that there were 522,913 live preterm births in the United States, corresponding to a 20% increase (from 10.6% to 12.7%) in the number of women delivering prematurely, when compared to 1990 (http://www.marchofdimes.com/peristats/). 

Babies born prematurely are at increased risk of having low birth weight (<2,500 grams) and of perinatal mortality [2]. While most deaths occur in babies born before 32 weeks of gestation, the biggest challenge arises when caring for those born at less than 28 weeks of gestational age. At this point, babies are considered to be of very low birth weight (VLBW), weighing <1,500 grams (3.33 lbs), and their organs are significantly less developed than those of those delivered at a later time. An estimated 6% of preterm babies are born before the completion of 28 weeks, while 71% are born between 34 and 36 weeks of gestation, and are referred to as “late preterm” [2–4]. 

The single most common cause of spontaneous PTB, aside from a previous premature delivery, is intrauterine infection in the mother (40%). Furthermore, the earlier on in the gestational time period at the time of delivery, the more likely it is that there is microbial infection in the amniotic cavity [5]. Seen especially in infants born before the completion of 34 weeks of gestation, amniotic fluid (AF) infection is often coupled with a proinflammatory response noted by the presence of cytokines such as tumor necrosis factor-alpha (TNF-*α*) and interleukin-6 (IL-6) [5]. High levels of inflammatory cytokines have also been found in the cerebral spinal fluid of preterm infants, but appear to be independent of gestational age [6]. Other risk factors for PTB include cervical or uterine abnormalities, lack of proper medical care, stress, smoking, and maternal age [1, 7]. 

In the clinical setting, inhibition of myometrial contractions (tocolysis) is the focus of therapeutic approaches for preventing PTB since the contracting uterus is the most commonly recognized precursor of an early delivery [8]. Magnesium sulfate (MgSO_4_) is currently the tocolytic agent of choice, used by approximately 70% of responding medical personnel, followed by terbutaline (13%), nifedipine (11%), and indomethacin (6%). Interestingly, none of the drugs mentioned above have been proven to have a significant effect in adequately delaying gestation or in enhancing neonatal outcome [9–12]. 

Currently unavailable, a tocolytic agent would require the widespread documentation of its efficacy and safety, for both the mother and fetus. In the United States, and also abroad, recent guidelines have stressed the importance of the need to explore the postnatal consequences of infants exposed to tocolytic agents in utero. It is here that the importance of nonclinical animal studies becomes evident as they are needed to investigate the findings which cannot be ethically, sufficiently, and/or safely determined with pediatric clinical trials [13–15].

## 2. The Pathway Leading to Preterm Birth

As mentioned previously, maternal infection is one of the leading causes of PTB, and is the only pathological process for which there is a direct causal relationship with prematurity [16]. However, despite a vast accumulation of human and animal data, the exact pathways and mechanisms leading to this point remain incompletely understood. 

While systemic maternal infections such as malaria and pneumonia can cause PTB, the occurrences of these infections are minimal in developed countries. Instead, the majority of infections leading to premature labor is often those which are intrauterine [17]. Unfortunately, the diagnosis of such types of infections early on in gestation is complicated. This can be attributed to the fact that the most tell-tale clinical signs of infection, which include fever, tenderness of the uterus, fetal tachycardia, and so forth, tend to occur late in the process of evolving infection, and do not present in a large percent of women, despite evidence that microorganisms are present in the amniotic cavity [18]. Mothers with infection of the amniotic cavity may not respond as well to treatment with tocolytics due to the presence of underlying inflammation [18, 19]. 

The most common route by which infection occurs is via the ascension of microorganisms from the vagina and cervix through the chorioamniotic membranes and into the amniotic cavity, where it will eventually cause infection in the fetus. As a result, a fetal inflammatory response may develop, most commonly denoted by funisitis (inflammation of the umbilical cord) which has been associated with the development of cerebral palsy [17, 20, 21]. 

The noted presence of bacteria and infection in the gestational compartment and other areas of pregnant women have been shown to trigger the immune system via cell surface recognition molecules such as toll-like receptors (TLRs) [22]. TLRs are transmembrane proteins capable of recognizing and responding to precise patterns of microbial components, and initiate the innate immune system in the host against nonself [23, 24]. 

Currently, 12 TLRs have been identified in mammals. In particular, TLR-2 recognizes Gram-positive bacteria while TLR-4 recognizes Gram-negative organisms and binds to lipopolysaccharide (LPS) [25–27]. LPS is a component of the outer wall of Gram-negative bacteria and is often used to stimulate an immune response in the animal model. Except for TLR-3, binding of mammalian TLRs activates the MyD88- (myeloid differentiation primary-response genes 88-) dependant pathway, which, in turn, activates the transcription factor nuclear factor-*κ* *β*  (*N* *F*-*κ* *β*). NF-*κ* *β* has been implicated in eliciting the expression of proinflammatory genes which play a role in PTB [24, 28, 29].

In humans, the binding of TLR-4 initiates a host inflammatory response involving cytokines such as IL-1 and TNF, thus playing a critical role in the pathogenesis of infection-associated PTB [30–32]. The inflammatory response is responsible for inducing steps in the latter part of the parturition cascade, such as decreased prostaglandin catabolism, functional progesterone withdrawal, increased expression of proteases, contraction-associated proteins, and increased uterine contractile activity, ultimately leading to labor and delivery [22].

## 3. Matrix Metalloproteinases

 During pregnancy, specific physiological processes such as cervical ripening, rupture of the fetal membranes, and placental detachment require the remodeling of the extracellular matrix (ECM) [33–37]. Rupturing of the membranes is believed to be the result of the effects of physical forces which are localized in areas surrounding the membranes that are made weaker by the degradation of structural collagens [38]. The matrix metalloproteinases (MMPs) are a family of enzymes (with more than 20 members identified) that use zinc-dependant catalysis to break down the components of the ECM [39], allowing for the movement of cells and tissue reorganization in order to support the growing fetus [40]. Several MMPs are constitutively generated by reproductive tissues, and a fluctuation in the gene expression level of certain MMPs has been observed during the various stages of parturition [41].

The ECM of the cervix, fetal membranes, placenta, and uterus are composed mainly of collagen types I and III. Investigators have shown that a remodeling of these collagens, mediated by the MMPs, may play a role in the pathway leading to birth [42]. A spontaneous rupturing of membranes before the completion of 37 weeks of gestation is considered to be of pathological origin and is a leading cause of PTB and delivery [43]. The MMPs are also believed to play a major role in the remodeling of the uteroplacental vasculature which changes throughout pregnancy [40]. 

Several lines of investigation have implicated specific MMPs in normal parturition as well as infection-triggered rupture of membranes and preterm birth. A cross-sectional study conducted by Maymon et al. [38] reported that, out of 353 women involved, concentrations of MMP-1 were detectable in 81.3% of amniotic fluid samples, with the concentrations increasing with gestational age. Additionally, preterm PROM was associated with a significant increase in the median concentrations of amniotic MMP-1 in both the presence and absence of infection, while term PROM was not associated with an increase in MMP-1 levels [38]. Interestingly, Fujimoto et al. found that a polymorphism resulting in increased MMP-1 promoter activity resulted in an increased risk of preterm premature rupture of the membranes [44]. In a cross-sectional study involving 365 subjects and conducted by Park et al. [45], increased levels of MMP-3 were associated with parturition, both term and preterm, as wells as microbial invasion of the amniotic cavity.

 Recently, much attention has been given to MMP-9, also known as 92-kDa type IV collagenase or gelatinase B [46]. MMP-9 is considered to be an enzyme which plays a role in the latter part of ECM remodeling and, unlike other MMPs, is produced and activated under certain conditions such as infection, active labor, and premature rupture of the membranes (PROM) [46, 47]. In the setting of intraamniotic infection, the concentrations of AF MMP-9 and the inflammatory cytokine IL-6 have been found to be significantly elevated [39]. A study conducted by Harirah et al. [39] reported that 22 out of 26 women with positive AF cultures had detectable levels of MMP-9, ranging from 30.1 to 541.9 ng/mL, while MMP-9 was nearly undetectable in women with negative AF cultures. The MMP-9 cutoff value used for predicting the presence or absence of infection was 13.6 ng/mL. In this study, spontaneous PTB was not explored as an outcome because, for the majority of women, the benefits of inducing labor or having a cesarean delivery outweighed those of going on with their pregnancies. 

In women with normal pregnancies, elevated levels of MMP-9 have been found in the cervicovaginal fluid and are associated with cervical ripening before labor, but are not a useful predictor for labor induction at term [48]. Under certain conditions, cervical ripening can be induced by the inflammatory process which involves the catabolism of the cervical ECM by enzymes discharged from infiltrating leukocytes [49]. Watari et al. [49] reported that, in a dose-dependent manner, the inflammatory cytokines TNF-*α* and IL-1, which have been associated with PTB, produce an increase in MMPs-1, -3, and -9 in a human uterine smooth muscle system. Interestingly, these MMPs are the only ones to have promoters that contain Activator Protein-1 and Ets-binding sites [49].

 While MMP-9 has been found to play a role in the events associated with term and preterm labor, comparable changes in MMP-2 protein levels and activity are often not reported. This may suggest that MMP-2 is expressed continuously throughout labor [46] while MMP-9 expression is induced by various factors as previously mentioned. On the other hand, Yonemoto et al. [37] have also reported, in 2005, on the gestational age-dependant increases in MMP-2 proenzyme activity and protein levels from the amnion in patients in labor, suggestive of the induction of enzyme expression later on in gestation. Moreover, Maymon et al. (2001) found a decrease in tissue inhibitor of matrix metalloproteinase-2 (TIMP-2) in association with term and preterm parturition, term and preterm rupture of fetal membranes, and microbial invasion of the amniotic cavity [50]. 

An increase in midtrimester concentrations of amniotic fluid MMP-8, IL-6, and angiogenin are risk factors for early spontaneous preterm delivery (<32 weeks). MMP-8 is released during neutrophil activation, and amniotic fluid concentrations are elevated both in patients with intraamniotic infection and in patients with negative amniotic fluid cultures who deliver preterm. Yoon et al. [51] have developed a rapid MMP-8 bedside test that predicts imminent preterm delivery. This test can be performed in 15 minutes and without laboratory equipment. In particular, an elevated MMP-8 level of >23 ng/mL is a powerful predictor of spontaneous preterm delivery (<32 weeks). Amniotic fluid studies can be used to improve the risk assessment for preterm delivery in women who undergo midtrimester amniocentesis for genetic indications [51]. 

Even though some of the best clinical predictors for PTL include cervical dilation >3 cm, vaginal bleeding, and ruptured membranes, these changes often appear too late for successful intervention [52]. However, the detection of plasma pro-MMP-9 levels and measurement of cervical length in combination with cutoff values of 65.157 ng/mL and 15 mm, respectively, are characterized by 90.9% sensitivity and 98.3% specificity and have been shown to possibly function as predictive factors for PTB and PTL within 7 days of presentation.

We have recently shown, using cDNA microarray technology, that several genes related to ECM homeostasis are upregulated in placental chorionic villi from patients who have labored as compared to patients who have undergone Cesarean deliveries without labor [53]. In particular, we found that MMP-1 was consistently elevated in placentas from patients who had labored as compared to placentas from patients who had not labored at both the mRNA and protein level. Interestingly, the upregulation in MMP-1 was seen in the fetal portion of the placenta, suggesting that the fetus can contribute to its own premature expulsion from a hostile intrauterine environment. 

**Figure 1:**
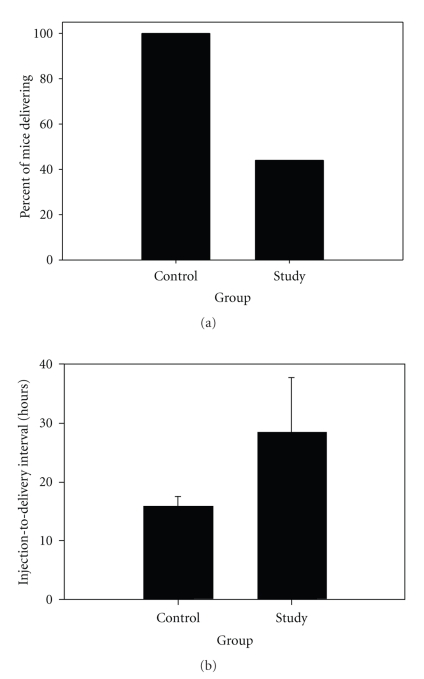
Effect of the MMP inhibitor GM6001 on preterm delivery in the mouse model. (a) Incidence of preterm delivery in the mouse model in either the absence (control group) or presence (study group) of GM6001 after i.p. 3.3 mg/kg LPS injection (*P* < .01, Fisher's exact test). (b) Change in LPS injection-to-delivery time interval in hours, in the control group versus study group (*P* < .005, Mann-Whitney test).

In 2007, we tested the nonspecific MMP inhibitor GM6001 (EMD Biosciences, La Jolla, CA) for its effect on endotoxin-triggered preterm labor in a mouse model [54]. GM6001 has *K*_*i*_ values for MMP-1, -2, -3, -8, and -9 which are in the nanomolar-to-picomolar range. Briefly, pregnant C57Bl/6 mice were injected with LPS on gestational days 15.5–16 in order to simulate infection-associated PTB and then received either GM6001 (study group) or vehicle (control group) 12 hours later. In the GM6001 group there was a significant decrease in the number of mice delivering prematurely when compared to the controls (Figure 1), suggesting that one or more of the MMPs are critical in the pathogenesis of infection-associated PTB.

## 4. The Endothelins

The human endothelin (ET) peptide family is made up of three distinct isoforms—ET-1, ET-2, and ET-3 [55]. Composed of 21 amino acid residues with two sets of intrachain disulfide linkages, the endothelins are produced in an array of tissues with different distribution patterns and regulate vasomotor tone, cell proliferation, and hormone production [55–57]. 

Two types of ET receptors, Endothelin-A (ET_A_) and Endothelin-B (ET_B_), have been identified in most mammalian species and belong to the superfamily of G-protein-coupled receptors (GPCRs) [58]. Even though their amino acid structures exhibit a significant amount of similar homology, the receptors have varying affinities for the three ET isoforms [58]. While the ET_A_ receptor has a much greater affinity for ET-1 as compared to ET-2 and ET-3, the ET_B_ receptor binds all three members with equal affinity [58]. 

 ET-1 is the most potent vasoconstrictor in the ET family, exhibiting a long duration of action, and is also the only member to be produced in endothelial cells [56]. ET can induce vasoconstriction via two major cell signaling systems: the opening of calcium (Ca^2+^) channels and phospholipase-C activation [56]. Additionally, ET-1 is also produced by a broad spectrum of cell types, including vascular smooth muscle cells, hepatocytes, breast epithelial cells, and astrocytes of the central nervous system [59].

The 203-amino acid residue peptide precursor preproendothelin-1 gives rise to ET-1 via the formation of the 38- or 39- (species-dependent) amino acid intermediate termed “big ET-1” which circulates in plasma [56]. Interestingly, big ET-1 is significantly less potent than ET-1 in terms of inducing contractile actions in vascular strips, but both forms are equally potent in raising blood pressure in vivo [57]. 

Endothelin-converting enzyme-1 (ECE-1) is responsible for the cleaving of big ET-1 between the Trp21 and Val22 residues, ultimately leading to the formation of ET-1 [56]. ET-1 is now implicated in an ever-growing list of pathologic processes, ranging from pulmonary hypertension to cancer. In particular, several lines of evidence support the involvement of the peptide in obstetrical disorders. An increase in maternal plasma ET-1 levels in patients with preeclampsia was first noted in 1990 [60]. Because the ET_A_ receptor has such a great affinity for ET-1, the possible therapeutic benefit of selective ET_A_ receptor antagonists in preeclamptic women needs to be further explored. Furthermore, ET_A_ receptor-directed therapy may overlap with antihypertensive management in preeclamptic patients. The usefulness of this approach remains uninvestigated [61]. 

 Several investigators found that, in pregnant rats, a chronic elevation in the serum levels of TNF-*α* and IL-6 and a reduction in uterine perfusion pressure lead to the activation of the ET system [62, 63]. Romero et al. found higher human amniotic fluid concentrations of ET-1, -2 among women who developed preterm labor and delivery and had culture-proven microbial invasion of the amniotic cavity [64] as compared to uninfected women, suggesting a role for the endothelins in infection-associated preterm birth. Mitchell et al. reported that ET-1 regulates prostaglandin production in human umbilical vein endothelial cells and cells derived from the amnion and decidua [65]. 

**Figure 2:**
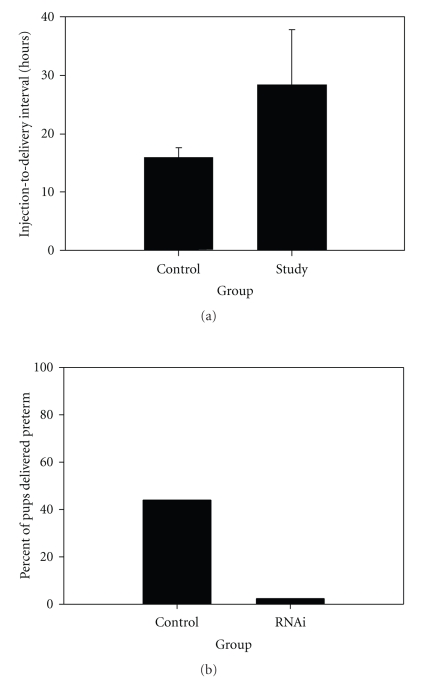
Effect of ET-1 blockade on preterm delivery in the mouse model. (a) Incidence of preterm delivery in the mouse model in the absence, presence of high-dose, or presence of low-dose BQ-123 (*P* < .01 between control and high-dose groups, Fisher's exact test). (b) Effect of SiRNA for ECE-1 in the animal model of infection-associated PTB (*P* < .001, Fisher's exact test).

We reported in 2004 that inhibition of endothelin-converting enzyme-1 (ECE-1), which colocalizes with ET-1 in the placenta [66], controls PTD in a mouse model of infection-associated PTL [67]. Subsequently, we have shown that ECE-1 is upregulated in our mouse model of PTL and that PTD is controlled with the ET_A_ receptor antagonist BQ-123 and with silencing of ECE-1 mRNA [68] (Figure 2).

Investigators have previously shown that, when compared to ET-1, the responsiveness of the rat uterus to the contractile effects of ET-3 is less than 1%, suggesting that the ET_A_ receptor is responsible for modulating uterine contractions [69]. Therefore, our group has recently developed a series of novel ET_A_ receptor antagonists. We have shown that one of our compounds, HJP-272, a 1, 3, 6-trisubstituted-2-carboxy-quinol-4-one (IC50, 70 nmol/L), is capable of significantly decreasing the number of pups that are dropped prematurely in the setting of infection [70]. 

While we do know that ET plays a very important role in the inflammatory cascade-associated infection-induced PTB, the use of ET_A_ receptor antagonists as a form of tocolytic therapy remains controversial. Bosentan, often used in the treatment of pulmonary hypertension, is a dual ET receptor antagonist approved for use in the United States. While the use of ET receptor antagonists may be useful for the treatment of pulmonary hypertension, these agents are often disregarded as potential tocolytics due to the fact that most are pregnancy Category X drugs.

## 5. The Link between ET-1 and the MMPs

Recently, we have tested whether endothelin-1 and MMP-1 act in the same molecular pathway in our mouse model of infection-associated preterm birth. We have shown that MMP-1 is upregulated in placental and uterine tissues of mice induced to preterm labor and delivery with LPS (manuscript submitted for publication) and that the MMP inhibitor GM6001 controls preterm birth in these same animal models. We also compared levels of MMP-1 in LPS-induced mice that were treated with RNAi directed at ECE-1 to levels of the protein in positive control LPS-induced mice with unaltered ET-1 systems. We were interested to find that knocking down ET-1 synthesis knocked down MMP-1 upregulation as well. Furthermore, those mice in which ECE-1 and, consequently, MMP-1 levels were reduced were largely prevented from developing preterm delivery (manuscript submitted).

Several lines of investigation in other biologic systems have established that ET-1 affects the synthesis of MMPs. Koyama and Tanaka [71] have recently shown that ET-1 stimulates the production of MMP-3 in cultured rat astrocytes. Similarly, Manacu et al. [72] found that, in primary cultures of human osteoarthritic chondrocytes, the presence of ET-1 in the medium triggered increased production of MMP-1 and MMP-13. Felx et al. [73] found that ET-1 promotes induction of MMP-2 and MMP-9 in human osteosarcoma cells via the transcription factor NF-*κ* *B*. Furthermore, treatment of these malignant cells with ET receptor antagonists decreases cell invasion. In ET-1-activated human optic nerve head astrocytes, levels of MMP-1, TIMP-1, and TIMP-2 are all increased. These changes are mediated through the ERK map kinases and protein kinase C [74]. ET-1 promotes cerebrovascular remodeling in type 2 diabetes through differential regulation of MMPs [75]. Murray et al. [76] produced a marked increase in MMP-2 activity and a significant decrease in collagen volume fraction in rat hearts by administering ET-1. More recently, they have shown that rats that underwent the surgical introduction of an aortocaval fistula had much less of a change in MMP-2 levels and in collagen volume fraction of the heart if they were treated with Bosentan, an endothelin receptor antagonist [77]. Interestingly, Lee et al. reported that vascular endothelial growth factor- (VEGF-) induced ET-1 production occurs via MMP-2 action, and independently of ECE-1 function [78]. Finally, Deschamps et al. [79] showed that the increase in MMP-1 levels in porcine hearts associated with ischemia and reperfusion injury did not occur if the animals were treated with the ET-1 receptor-A antagonist BQ-123.

## 6. Conclusion

**Figure 3:**
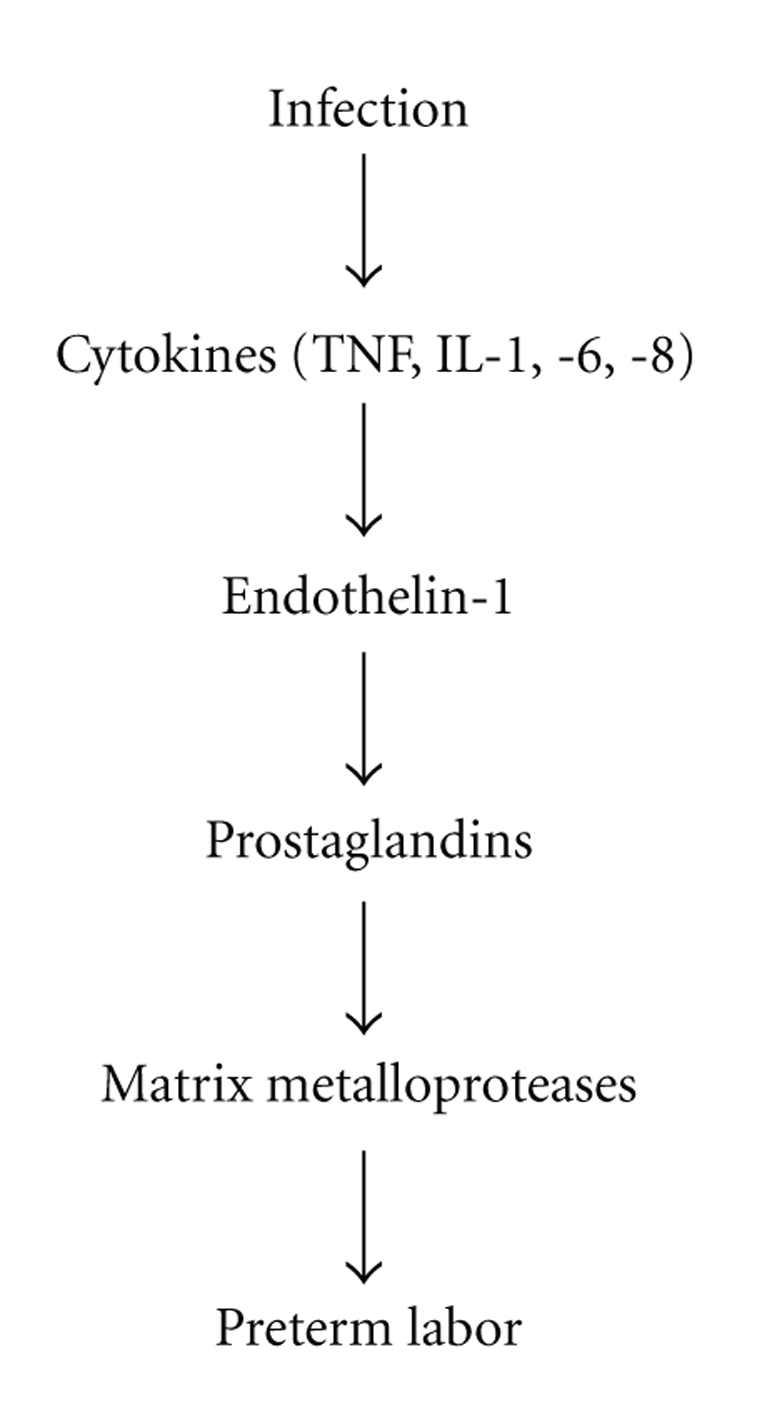
Parturition cascade with putative sites of action of endothelin-1 and matrix metalloproteinases.

Increased levels of inflammatory cytokines in the setting of infection ultimately lead to PTL and PTB via several different mechanisms and feedback loops which occur at the maternal fetal interface. Several lines of evidence indicate that both ET-1 and the MMPs are critical parts of the molecular pathway that connects ascending bacterial infection to PTB. A simple skeletal version of the parturition cascade, including the putative sites of activity of ET-1 and the MMPs, is shown in Figure 3. On one hand, the risk of teratogenicity caused by any potential tocolytic agent is minimized, because the period of administration is transient, and because the agent would be given well after organogenesis is complete. On the other hand, as both endothelin and the MMPs play a role in embryonic and fetal development, agents blocking the actions of either of these important molecular players are somewhat suspect for potential fetal toxicity. A better understanding of the infection-associated preterm labor molecular pathway is needed, so that more and more potential targets for therapy can be revealed.
